# Percutaneous-perventricular device closure of ventricular septal defect: mid-term follow-up

**DOI:** 10.1186/s12893-020-00854-0

**Published:** 2020-09-18

**Authors:** Long Wang, Lin Xie, Weiqiang Ruan, Tao Li, Changping Gan, Ke Lin

**Affiliations:** Department of Cardiovascular Surgery, West China Hospital, Sichuan University, No.37 Guoxue Lane, Wuhou District, Chengdu, Sichuan 610041 People’s Republic of China

**Keywords:** Ventricular septal defect, Minimally invasive surgery

## Abstract

**Background:**

This report presents updated data and mid-term follow-up information to a former study introducing the novel technique of percutaneous-perventricular device closure of doubly committed subarterial ventricular septal defect.

**Methods:**

Thirty-eight patients were added to the former series. There were 54 patients in total who had isolated doubly committed subarterial ventricular septal defects and underwent percutaneous-perventricular device closure. Closure outcomes and possible complications were measured in the hospital and during the 2.5-year follow-up.

**Results:**

Surgery was successful in 53 patients (98.1%). There was no death, residual shunt, new valve regurgitation or arrhythmia either perioperatively or during the entire follow-up period. Only one patient developed pericardial effusion and tamponade in the former series. The mean hospital stay was 3.2 ± 0.6 days (range, 3.0 to 6.0 days), and only one unsuccessful case needed blood transfusion (1.9%).

**Conclusions:**

The percutaneous-perventricular device closure of isolated doubly committed subarterial ventricular septal defects appeared to be safe. Close monitoring for bleeding is essential postoperatively, especially in younger patients. This technique is generally safe with acceptable mid-term follow-up.

## Background

Doubly committed subarterial ventricular septal defect (VSD) is a unique type of VSD accounting for about 5–7% of all VSDs [1], which has been a contraindication for percutaneous transcatheter device closure due to the challenging geometry consisting of the upper edge of the defect and the aortic valve. Perventricular device closure of VSD without cardiopulmonary bypass (CPB) under transesophageal echocardiography (TEE) guidance has been widely practiced in China [2, 3], and was also used in some cases in Europe [4]. This technique has also been introduced recently in the treatment of doubly committed subarterial VSD with encouraging initial results and excellent cosmetic outcome [5–7]. Compared to a mini-thoracotomy, we have advanced a novel and more minimally invasive method by combining perventricular device closure with the percutaneous approach [8]. We successfully closed doubly committed subarterial VSDs through a pinhole-size puncture on the chest. The short-term result has been introduced elsewhere [8], and we present an update of the patient series and its mid-term follow-up result.

## Methods

In our previous report [8], patients were enrolled from January to May 2015, 16 patients (9 male) were included; In this update, from January 2015 to January 2019, patients who presented with isolated doubly committed subarterial VSDs diagnosed by transthoracic echocardiography (TTE) were added to this pool. The inclusion and exclusion criteria were consistent with the previous study [8]. Again, individual informed consent was obtained from the adult patients and both parents of all the pediatric patients [8]. All patients underwent 12-lead electrocardiography (ECG) before the surgery and detailed TEE under general anesthesia before the procedure by the same echocardiographer. The maximum diameter of the defect as assessed by multiple views, the presences of any aortic or pulmonary regurgitation, and the presence of any major coronary branch crossing the infundibulum of right ventricle (RV) and any preoperative arrhythmia were recorded [8].

The general principles have been described elsewhere and in the previous study [8, 9]. However, an improvement in terms of technical detail was made in the updated population. In the previously described method, after retrieving the cable of the first eccentric device, the delivery sheath would be kept inside the RV. That is to say, the tip of the sheath was kept in the RV cavity. In the revised method, instead of keeping the depth of the sheath carefully, the sheath would be advanced prophylactically into the pulmonary artery for 2–3 cm after removing the cable. When the second device was ready, the sheath would be pulled back to the RV in order to deploy the right disc of the device against the RV free wall.

In the patients in the updated study, continuous TTE was replaced by an intermittent check every 4 h. Patients also received TTE examination and 12-lead ECG on the second postoperative day, the day before discharge and during the follow-up period [8]. The position and stability of the device, residual shunt, aortic or pulmonary regurgitation, tricuspid regurgitation, RV outflow tract patency, as well as any arrhythmia were carefully checked during the examinations [8].

### Statistics

The data for nominal variables were expressed as percentages and continuous variables were expressed by mean ± SD and/or median (range). SPSS 16.0 for Windows (SPSS Inc., Chicago, IL, USA) was used for statistical analysis. The data not applicable to comparable analysis were descriptively reported.

## Results

Thirty-eight patients were added to the update, so a total of 54 patients (26 male) were included according to the selection criteria [8]. The mean age was 5.0 ± 6.6 years (range, 0.6 to 30.0 years) and the mean body weight was 18.7 ± 12.8 kg (range, 7.8 to 55.0 kg) in the series. The mean VSD diameter measured by preoperative TTE was 4.4 ± 1.4 mm (range, 2.0 to 5.5 mm) and 4.4 ± 1.1 mm (range, 3.0 to 7.0 mm) by TEE after general anesthesia (Table 1). Before the procedure, 10 patients (18.5%) had trivial aortic regurgitation without aortic valve prolapse, and five patients (9.3%) had trivial tricuspid regurgitation. One patient was found with trivial pulmonary regurgitation (1.9%) preoperatively.

**Table 1:** Characteristics of study patients

Sex (male/female)	26/28
Mean age at time of procedure (years)	5.0 ± 6.6 (range, 0.6–14.0)
Mean body weight (kg)	18.7 ± 12.8 (range, 7.8–40.0)
Mean VSD diameter by TTE (mm)	4.4 ± 1.4 (range, 2.0–5.5)
Mean VSD diameter by TEE (mm)	4.4 ± 1.1 (range, 3.0–7.0)
Mean eccentric occluder diameter (mm)	7.1 ± 1.3 (range, 5.0–10.0)
Preoperative arrhythmia	
Atrioventricular block	0 (0%)
Right bundle branch block	0 (0%)
Other arrhythmia	0 (0%)
Preoperative valve regurgitation	
Aortic regurgitation	10 trivial (18.5%)
Pulmonary regurgitation	1 (1.9%)
Tricuspid regurgitation	5 trivial (9.3%)

Regarding the outcomes of percutaneous perventricular device closure, 53 patients completed the percutaneous perventricular device closure successfully (53/54, 98.1%). The mean size of the device for VSD closure was 7.1 ± 1.3 mm (range, 5.0 to 10.0 mm), and the device size of diameter for the RV tunnel was 5.0 mm or 6.0 mm. No death, residual shunt, device dislocation or obstruction of the RV outflow tract was observed in the patients in the updated study (Table 1). Fifty-four patients were followed up for 3 months, while 49 patients for 6 months, 41 for 1 year, 32 for 2 years, 24 for 3 years, and 13 for 4 years (Table 2).

**Table 2:** Follow-up outcomes of percutaneous perventricular device closure

In-Hospital / Follow-Up	Death	Residual Shun	RVOT Obstruction	Valve Regurgitation n/n (%)		
	n/n(%)	n/n(%)	n/n(%)	AR	PR	TR
After procedure in the OR	0/54 (0%)	4/54 (7.4%)	0/54 (0%)	1/54 (1.9%)	0/54 (0%)	3/54 (0%)
Discharge	0/54 (0%)	0/54 (0%)	0/54 (0%)	0/54 (0%)	0/54 (0%)	0/54 (0%)
3 months	0/54 (0%)	0/54 (0%)	0/54 (0%)	1/54 (1.9%)	0/54 (0%)	1/54 (0%)
6 months	0/49 (0%)	0/49 (0%)	0/49 (0%)	1/49 (2.0%)	0/49 (0%)	1/49 (0%)
1 years	0/41 (0%)	0/41 (0%)	0/41 (0%)	1/41 (2.4%)	0/41 (0%)	1/41 (0%)
2 years	0/32 (0%)	0/32 (0%)	0/32 (0%)	1/32 (3.1%)	0/32 (0%)	0/32 (0%)
3 years	0/24 (0%)	0/24 (0%)	0/24 (0%)	0/24 (0%)	0/24 (0%)	0/24 (0%)
4 years	0/13 (0%)	0/13 (0%)	0/13 (0%)	0/13 (0%)	0/13 (0%)	0/13 (0%)

*AR* Aortic regurgitation, *OR* Operating room, *PR* Pulmonary regurgitation, *RVOT* Right ventricular outflow tract, *TR* Tricuspid regurgitation

**Fig. 1:**
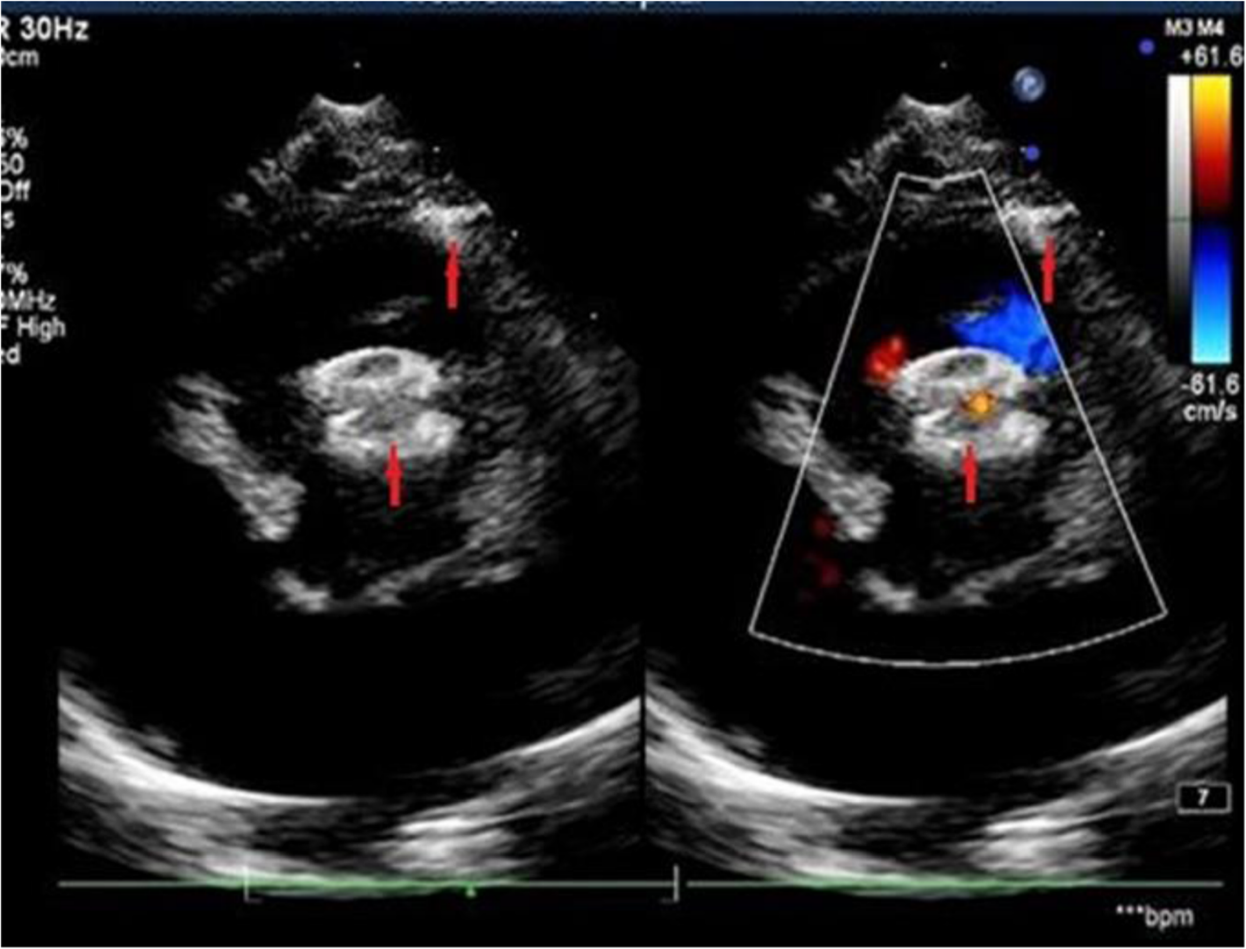
Red arrow indicates the concentric device was deployed in the right ventricular outflow tract

No new findings were observed in the updated patients in terms of new postoperative aortic, pulmonary or tricuspid valve problems or any new onset of arrhythmia (Table 1). From TTE clinical follow-up, there was no acceleration of blood flow in the RVOT (Fig. 1).

Preoperative aortic regurgitation in 9 patients had disappeared during the follow-up period, with one case which remained stable during the follow-up periods. Device-related aortic regurgitation was not found. Thrombosis, hemolysis, infective endocarditis, or conduction abnormalities were not encountered, other than the case in which bleeding occurred in one patient due to undesired dislodgement of the delivery sheath [8]. This case was regarded as unsuccessful. In another two cases in the updated study, a small amount of pericardial effusion was found after several hours in the ICU (maximal depth: 8 mm and 1.0 cm respectively). A single-lumen central venous catheter was used to drain the pericardial cavity and the effusion did not increase.

The postoperative recovery was smooth. The mean postoperative hospital stay was 3.2 ± 2.5 days (range, 2.0 to 6.0 days). The second unsuccessful case received blood transfusion in the previous report [8].

## Discussion

The application of perventricular device closure has brought selected patients with doubly committed subarterial VSD to a minimally invasive era with reasonable outcomes [6, 7]. Furthermore, we have introduced the so called percutaneous-perventricular device closure technique in our previous publication combining percutaneous transcatheter device closure with perventricular device closure, which has dramatically reduced the surgical site from a small incision to a pinhole [8]. Based on a relatively small series, the success rate was high with only one case failing due to pericardial effusion in the operating room. In the follow-up period, no other major problems were found. However, only 16 patients were included in that series, and the follow-up period was only 1 year. In order to investigate the effectiveness and safety of this novel technique, we have updated the sample size and continued focusing on outcomes for a longer period of time.

Not surprisingly, taking the complete and successful rate of VSD closure into consideration, the effectiveness of this technique remained. All the VSD devices have been successfully placed without any residual shunting or device dislocation. These devices did not cause any new valve or rhythm problem even with a longer follow-up period. This was quite consistent with those studies using perventricular technique alone [5–7, 10]. Acute pericardial effusion was found in one case due to dislodgement of the sheath from the RV in the previous report [8], and this event was explained as an “accident” by us then. In our old technique protocol, after releasing the VSD device, the tip of sheath should be kept right in the RV outflow tract without compromising the first device or dislodging out of the heart. This is actually difficult and very demanding due to the small distance between the device and the RV free wall. Technical improvements have been made in the update to avoid this pitfall. By parking the sheath in the pulmonary artery temporarily before deploying the second hemostatic device, a reasonable length of sheath could be kept safely in the heart, Thus, no more dislodgement was found in any of the 38 patients in the updated study.

At the same time, pericardial effusion was noticed in two additional patients in the Intensive Care Unit after uneventful surgeries. The effusion was drained and caused no further adverse results, but still caught our attention. These two patients were aged 7 and 8 months respectively, so we assumed that their RV free wall in the puncture region was thin. The hemostatic device (Shanghai Shape Memory Alloy Material Co. Ltd., Shanghai, China) has a 5 mm high waist, which might be greater than the myocardium. Delayed bleeding or oozing could occur from the less packed “sandwich”. This complication implied that careful monitoring for the pericardial effusion might still be needed after an uncomplicated surgery, especially in younger patients less than 1 year old. Additionally, an internal mammary artery injury occurred in a patient aged one in the updated series. This might be due to a variant course of the artery, where the artery was closer to the edge of the sternum. Despite this, we still believe that the puncture point should be located adjacent to the left border of the sternum.

As compared to valve problems or arrhythmia, an obstruction of the RV outflow tract might be more technique specific and of interest in the follow-up. We have expanded the study population and observed every patient for 3 years. No cases presented with RV outflow tract obstruction in the whole follow-up period, not even in infants. No obvious flow acceleration was noticed in any echocardiographic exam. We did not measure geometric numbers of the RV outflow tract in any patient before the surgery. However, it is understandable that patients with VSD, especially a doubly committed subarterial one, would have a wider or larger infundibular region. This characteristic might allow a more crowded outflow tract without flow acceleration.

## Conclusions

In summary, with a larger study population size and a longer follow-up period of time, the percutaneous-perventricular device closure of doubly committed subarterial VSDs still appeared to be an extremely minimally invasive and effective technique. Although with improvements in the technique, the most common complication is still bleeding. Close monitoring is very necessary postoperatively no matter how uncomplicated the procedure is, and special attention should be paid to younger patients. Other than that, this technique is generally safe with acceptable mid-term outcomes.

## Data Availability

The datasets used and/or analysed during the current study are available from the corresponding author on reasonable request.

## Abbreviations

VSD: Ventricular septal defect
CPB: Cardiopulmonary bypass
ECG: Electrocardiography
TEE: Transesophageal echocardiography
TTE: Transthoracic echocardiography
AR: Aortic regurgitation
AVB: Atrioventricular block
OR: Operating room
PR: Pulmonary regurgitation
RVOT: Right ventricular outflow tract
TR: Tricuspid regurgitation
